# A Rigid–Flexible Coupled Six-Dimensional Force Sensor and Its PINN-Based Decoupling Algorithm

**DOI:** 10.3390/s26072038

**Published:** 2026-03-25

**Authors:** Yinlong Zhu, Zhengyu Xie, Chuanwei Lu, Shuang Xi, Xu Wang

**Affiliations:** 1 College of Mechanical and Electronic Engineering, Nanjing Forestry University, Nanjing 210037, China; ylzhu@njfu.edu.cn (Y.Z.); xzywlkq@njfu.edu.cn (Z.X.); shuangxi@njfu.edu.cn (S.X.); 2 College of Chemical Engineering, Nanjing Forestry University, Nanjing 210037, China; hg@njfu.edu.cn

**Keywords:** six-dimensional force sensor, structural decoupling, sensor decoupling algorithm, PINN

## Abstract

Six-dimensional force sensors are widely used in compliant robotic control and safe human–machine interactions due to their mature sensing mechanisms and high accuracy. However, conventional six-dimensional force sensors often suffer from complex structures, bulky size, and high manufacturing costs. To address these limitations, this paper proposes a compact and low-cost six-axis force sensor based on capacitive sensing. By employing a tailored arrangement of flexible sensing units, partial structural decoupling of force and torque in specific directions is achieved. A Physically Informed Neural Network (PINN) is further introduced to decouple the residual coupled signals. Experimental results demonstrate that the proposed method significantly improves decoupling accuracy, achieving force decoupling errors of 1.75%, 1.20%, and 1.31% for *F_x_*, *F_y_*, and *F_z_*, respectively, and torque decoupling errors of 0.95%, 0.93%, and 0.97% for *M_x_*, *M_y_*, and *M_z_*. The proposed sensor offers low-cost fabrication, compact integration, and high sensitivity, making it well suited for lightweight and high-precision sensing applications.

## 1. Introduction

With the rapid advancement of intelligent robotics, the demand for effective human–robot interaction has grown steadily [1,2,3]. Although visual sensing can provide object pose information and partial physical states, it fails to accurately capture interaction forces. As a result, six-dimensional force sensors have become an essential complement in human–robot interaction systems [4]. By simultaneously measuring three-dimensional forces and torques, these sensors provide critical feedback for stable interaction and precise control in intelligent robotic applications [5].

Traditional six-axis force sensors can generally be classified into two categories: cross-beam structure sensors [6] and parallel structure sensors [7]. Cross-beam sensors typically consist of a central body with multiple radially distributed cantilever beams (commonly four or eight), on which strain gauges are mounted. Owing to their mature design, compact structure, high stiffness, and excellent dynamic response, such sensors have been widely adopted. However, their complex mechanical structures and demanding fabrication processes lead to relatively high manufacturing costs [8]. Parallel-structure force sensors are composed of upper and lower platforms connected by multiple independent load-bearing legs, each integrating a unidirectional force sensor. This configuration provides high load capacity, good structural rigidity, and isotropic force sensing performance. Nevertheless, the signal decoupling process is typically complex. The Stewart platform sensor [9] is a representative example of this category and enables force measurement over a wide range. However, the large number of mechanical components may introduce structural instability and susceptibility to overload [10], which ultimately constrains its practical measurement performance.

Unlike strain-gauge-based sensors, capacitive sensors offer inherently high sensitivity and resolution [11]. Their relatively simple structures facilitate miniaturization and lightweight design [12,13], while also reducing manufacturing costs [14]. In practical applications, capacitive six-axis force sensors exhibit good durability, environmental adaptability, and cost-effectiveness. Recent studies have demonstrated the feasibility and advantages of capacitive six-dimensional force sensing. Tan et al. [1] proposed an S-shaped beam combined with a comb-type capacitive array, achieving high sensitivity and low crosstalk, with decoupling errors below 2.59% of full scale in all directions. Mao et al. [15] developed an ultra-lightweight and compact high-precision sensor using flexible structures and thermosensitive materials, achieving a fast response time of 90 ms. Choi et al. [16] introduced a dual-layer rigid PCB with a silicone pillar array, balancing low cost and high robustness, and enabling force detection ranges of 0–10 N (*F_x_*, *F_y_*), shear force up to 4 N, and torque measurement (*M_x_*, *M_y_*) up to 4 N·m. These works demonstrate that capacitive six-axis force sensors provide an effective solution for achieving high-precision, decoupled multi-axis force and torque measurement.

Traditional decoupling algorithms for multi-axis force sensors mainly include polynomial regression [17] and backpropagation (BP) neural networks [18]. Polynomial regression can model nonlinear coupling relationships but suffers from dimensional explosion and overfitting in high-dimensional, strongly coupled systems. BP neural networks offer strong nonlinear fitting capability; however, they are highly dependent on large, high-quality datasets and lack explicit physical constraints, which limits their robustness. Several hybrid and optimized learning-based approaches have been reported. Wang et al. [19] proposed a two-stage framework combining polynomial regression and neural networks, achieving an 87.80% classification accuracy with root mean square errors of 5.63% for force and 4.82% for torque. Zha et al. [20] optimized BP neural networks using genetic algorithms, reducing relative errors to below 0.85%. Chen et al. [21] further improved Extreme Learning Machines (ELMs) with whale optimization algorithms to enhance decoupling accuracy.

Although these methods can achieve low decoupling errors with sufficient training data, they typically require extensive calibration datasets and lack physical interpretability, making them prone to overfitting under limited or noisy data conditions. To address these limitations, this paper introduces a Physically Informed Neural Network (PINN), in which governing physical equations are embedded into the loss function. By incorporating prior physical knowledge, the proposed approach achieves high decoupling accuracy with small sample sizes, while ensuring physically consistent predictions, thereby significantly improving robustness and generalization in practical sensing applications.

This paper proposes a six-dimensional capacitive force sensor based on capacitance variation principles, featuring a rigid–flexible coupled structure and a Physically Informed Neural Networks (PINNs)-based decoupling algorithm. Through rational partitioning of sensing units and optimized hardware layout, the proposed sensor achieves effective structural pre-decoupling, significantly reducing cross-coupling among force and torque components. In particular, the sensor demonstrates superior performance in separating tangential forces from normal forces, as well as normal forces from torques. The structural pre-decoupling of coupled loads substantially improves the efficiency of the subsequent decoupling process and reduces computational complexity. Based on the preliminary decoupling derived from multi-channel capacitance variations, a PINN-based decoupling model is trained to further suppress residual coupling and predict accurate force and torque components under complex loading conditions. Furthermore, the proposed sensor was integrated into a multifunctional robotic arm and validated through grasping and manipulation experiments in realistic operating environments, demonstrating reliable performance for small-scale robotic grasping and handling applications.

## 2. Sensor Structure Design and Fabrication

### 2.1. Structural Design

**Figure 1:**
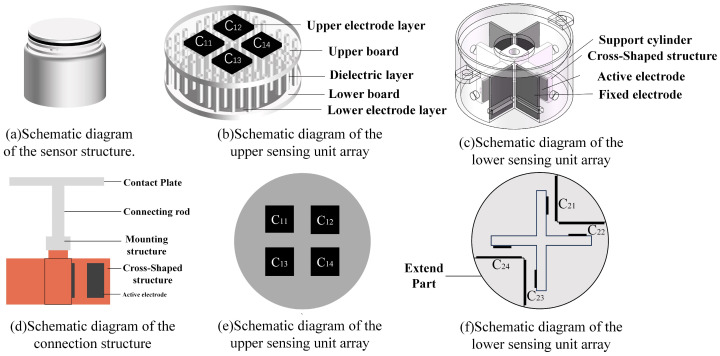
Sensor structure diagram.

As illustrated in Figure 1a, the proposed sensor integrates two groups of capacitive sensing units within a unified structure. Figure 1b shows the upper layer consists of four horizontally arranged flexible sensing units designed for the detection of *M_x_*, *M_y_*, and *F_z_*. This unit includes a circular common electrode with a diameter of 22 mm and four square electrodes, each with a side length of 5 mm. The electrodes are mechanically interconnected through micro-cylindrical dielectric structures with a diameter of 1 mm and a height of 2 mm. As illustrated in Figure 1e four capacitive sensing elements denoted as C_11_, C_12_, C_13_, and C_14_. As illustrated in Figure 1c. The lower layer comprises four vertically oriented capacitive sensing units responsible for measuring *F_x_, F_y_*, and *M_z_*. In each sensor unit, a rectangular fixed electrode which is 10 mm × 6 mm is attached to the inner wall of the supporting cylinder, while a movable electrode which is 4 mm × 3 mm is mounted on a cross-shaped electrode bracket. The corresponding capacitive sensing elements in this layer are defined as C_21_, C_22_, C_23_, and C_24_, as is shown in Figure 1f. The rigid mechanical framework of the sensor consists of a top contact plate, a connecting structure, and a support cylinder. The support cylinder is designed as a hollow cylindrical structure with an outer diameter of 23 mm, a wall thickness of 1 mm, and internal protrusions to facilitate structural integration and electrode positioning. The connection structure between the two sensing units is illustrated in Figure 1d.

**Figure 2:**
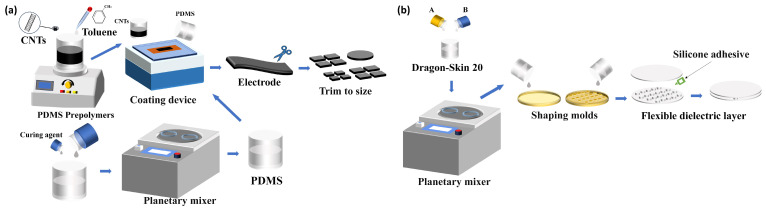
Sensor fabrication process: (**a**) electrode preparation process; (**b**) dielectric layer preparation process.

The electrodes were fabricated using carbon nanotubes (CNTs) with a purity of 95% (CNTs Xinfeng Nanotechnology Co., Ltd., Nanjing, China), with lengths ranging from 10–30 µm and diameters of 10–20 nm. CNTs were selected as the electrode material owing to their excellent flexibility, high electrical conductivity, and good mechanical compliance with flexible substrates. The interconnected CNT network structure can effectively accommodate mechanical deformation while maintaining continuous conductive pathways. As a result, the electrode can maintain stable and reliable electrical performance under repeated mechanical loading, making it highly suitable for flexible sensing applications. Polydimethylsiloxane (PDMS, Dow Inc., Midland, MI, USA) was used as the adhesive layer material, while the dielectric layer was composed of silicone rubber (Dragon Skin 00-20, Smooth-On, Inc., Macungie, PA, USA). The electrode layer was prepared via a coating–adsorption process. The dielectric layer was fabricated by thoroughly mixing components A and B of Dragon Skin 00-20, followed by mold injection and thermal curing. The detailed fabrication procedure is presented in Figure 2.

### 2.2. Fabrication of the Sensor

**Figure 3:**
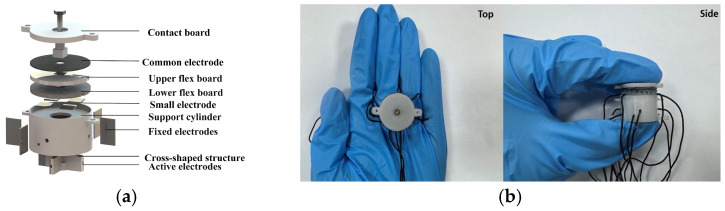
(**a**) Assembly of sensor; (**b**) photographs of actual sensor.

As shown in Figure 3a, the upper sensing unit adopts a multilayer configuration consisting of a common electrode, upper and lower flexible electrode plates, and an intermediate dielectric layer. In the lower sensing unit, the fixed electrode is attached to the inner wall of the support cylinder, whereas the movable electrode is mounted on a cross-shaped support structure. The contact plate passes through the upper electrode array and is rigidly connected to both the cross-shaped structure and the top of the support cylinder, as illustrated in Figure 3b.

## 3. Sensor Operating Principles and Decoupling Methods

### 3.1. Principle of Sensor Operation

**Figure 4:**
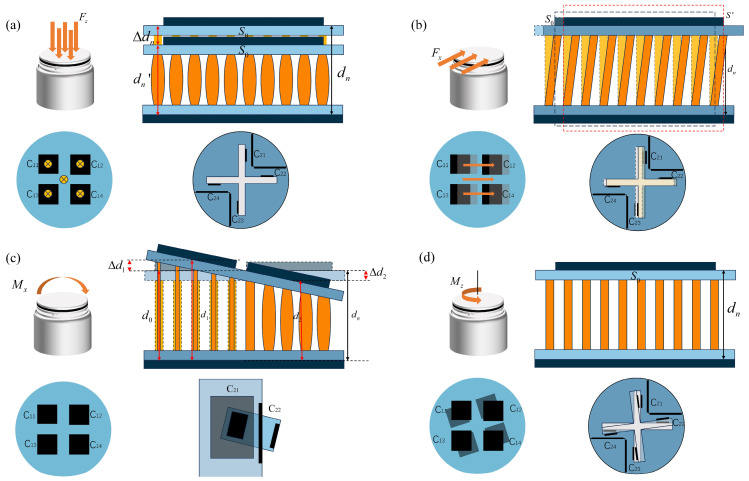
Schematic diagrams of sensor operation. (**a**) Axial force *F*_z_ sensing decoupling schematic. (**b**) Tangential force *F*_x_ sensing decoupling schematic. (**c**) Moment *M*_x_ sensing decoupling schematic. (**d**) Moment *M*_z_ sensing decoupling schematic.

Figure 4a illustrates the response under a normal force *F_z_*. In this case, the electrode spacing in the upper sensing unit decreases, leading to an increase in the corresponding capacitance values, while the lower sensing unit remains essentially unaffected. Figure 4b presents the effect of a tangential force *F_x_*. The electrode spacing of C_21_ in the lower sensing unit decreases, whereas that of C_23_ increases, resulting in opposite capacitance variations. Under the action of *F_y_*, a symmetric variation pattern is observed in the orthogonal sensing elements. The influence of bending moments *M_x_* and *M_y_* is illustrated in Figure 4c. The upper sensing unit undergoes differential deformation, characterized by reduced electrode spacing on one side and increased spacing on the opposite side. This asymmetric structural response produces differential capacitance changes among the upper sensing elements. Figure 4d depicts the case of torsional moment *M_z_*. In this condition, electrode spacing variation occurs primarily within the lower sensing unit, enabling the identification of *M_z_* through the corresponding capacitance changes.

### 3.2. Decoupling Methods for Sensors

To more clearly describe the relationship between the six-dimensional forces and moments and the capacitive output of the sensor, we assume that the capacitance of the sensor output is represented as C = (C*_Fx_*, C*_Fy_*, C*_Fz_*, C*_Mx_*, C*_My_*, C*_Mz_*)ᵀ, where the six components denote the capacitive signals produced by the sensor in response to the applied forces and moments across the six dimensions. Ideally, six-dimensional force sensors are designed to output distinct signals for each degree of force. However, due to the structural characteristics of typical parallel plate sensors, the output signals for each dimension may exhibit some degree of interdependence. To address this issue, it is essential to investigate the coupling between the force and moment measurements of the sensor and to propose a specific, precise, and acceptable decoupling strategy aimed at enhancing measurement accuracy and establishing a reliable one-to-one relationship between input and output.

#### 3.2.1. Structural Decoupling of Sensors

By integrating flexible electrodes with a rigid supporting frame, structural stability and uniform deformation within each capacitive sensing unit are ensured. Under all loading conditions, the effective area of the common electrode in the upper sensing unit fully covers the lower electrode array. As a result, when tangential forces *F_x_*, *F_y_*, or torsional moment *M_z_* are applied, neither the electrode overlap area nor the inter-electrode spacing of C_11_–C_14_ undergoes significant variation, and their capacitance values remain essentially unchanged.

Similarly, in the lower sensing unit, the fixed electrodes consistently encompass the movable electrode regions. Consequently, under the application of normal force Fz, the electrode overlap area of C_21_–C_24_ remains constant, and the resulting capacitance variation is negligible.

Through this structural configuration, preliminary mechanical decoupling of capacitance responses with respect to different load components is achieved. The corresponding load–capacitance relationships are summarized in Table 1.

From the table, we can deduce that the mathematical model relating force, moment, and capacitance in each dimension is as follows: the equivalent capacitance variations induced by the six-dimensional loads (*F_x_*, *F_y_*, *F_z_*, *M_x_*, *M_y_*, *M_z_*) are denoted as C*_Fx_*, C*_Fy_*, C*_Fz_*, C*_Mx_*, C*_My_*, and C*_Mz_*. Meanwhile, the capacitance variations of the individual sensing units are expressed as ΔC_11_, ΔC_12_, ΔC_13_, ΔC_14_, ΔC_21_, ΔC_22_, ΔC_23_, and ΔC_24_, in accordance with the decoupling principle of the six-dimensional force sensor mentioned above.

(1) $$\left\{\begin{array}{c} C_{F_{x}}=\left(\Delta C_{21}-\Delta C_{23}\right)/2\\ C_{F_{y}}=\left(\Delta C_{22}-\Delta C_{24}\right)/2\\ C_{F_{z}}=\left(\Delta C_{11}+\Delta C_{12}+\Delta C_{13}+\Delta C_{14}\right)/4\\ C_{M_{x}}=\left[\begin{array}{cc} \left(-\Delta C_{11}-\Delta C_{12}+\Delta C_{13}+\Delta C_{14}\right)/4 & \left(\Delta C_{22}-\Delta C_{24}\right)/2 \end{array}\right]\\ C_{M_{y}}=\left[\begin{array}{cc} \left(\Delta C_{11}+\Delta C_{13}-\Delta C_{12}-\Delta C_{14}\right)/4 & \left(\Delta C_{21}-\Delta C_{23}\right)/2 \end{array}\right]\\ C_{M_{z}}=\left(\Delta C_{21}+\Delta C_{23}-\Delta C_{22}-\Delta C_{24}\right)/4 \end{array}\right.$$

Although the proposed structural design enables partial decoupling, it cannot completely separate the effects of *M_x_*-*F_y_* and *M_y_*-*F_x_*. Therefore, an additional decoupling algorithm is required to achieve more accurate separation of these moment components.

#### 3.2.2. Six-Dimensional Force Decoupling Method Based on PINN

The Yeoh hyperelastic model is adopted due to its capability to accurately capture the nonlinear deformation behavior of elastomer materials while maintaining a relatively simple formulation. Accordingly, the strain energy density function of the Yeoh model is expressed as:

(2) $$W=\sum_{i=1}^{3}C_{i0}{\left(I_{1}-3\right)}^{i}+\sum_{i=1}^{3}\frac{1}{D_{i}}{\left(J-1\right)}^{2i}$$

where W denotes the strain energy density, $C_{i0}$ and $D_{i}$ are material parameters of the Yeoh model, $I_{1}$ is the first invariant of the deformation tensor, and J represents the determinant of the deformation gradient, which describes the volume change of the material.

In previous work, the constitutive parameters of the flexible material were characterized via uniaxial tensile experiments. Under normal loading, the true principal stresses $\sigma_{i}$ (i=1,2,3) are obtained by differentiating the strain energy density function with respect to the principal stretch ratios $\lambda_{i}$, expressed as:

(3) $$\sigma_{3}=2(\lambda_{3}^{2}-1/\lambda_{3})\left\{C_{10}+2C_{20}{\left[2/\lambda_{3}+\left(\lambda_{3}^{2}-3\right)\right]}^{2}\right\}$$

where $\sigma_{3}$ denotes the principal stress along the loading direction and $\lambda_{3}$ is the corresponding principal stretch ratio.

Based on the stress definition σ=F/S, the relationship between the applied normal force and the resulting capacitance variation can be derived as:

(4) $$F_{z}=2S_{0}\left(C_{F_{z}}/C_{0}+1\right)\left[{1/\left(C_{F_{z}}/C_{0}+1\right)}^{2}-C_{F_{z}}/C_{0}-1\right]\left\{C_{10}+2C_{20}{\left[2C_{F_{z}}/C_{0}+\frac{1}{{C_{F_{z}}/C_{0}}^{2}}-1\right]}^{2}\right\}$$

When the sensor is subjected to a tangential force $F_{x}$, each microcylindrical structure can be approximated as a vertical rod with height *h* and cross-sectional area A. The resulting shear strain is expressed as $\gamma =\delta_{t}/h$, where $\delta_{t}$ denotes the tangential displacement of the upper plate. The corresponding shear stress can therefore be written as:

(5) $$\tau =G\gamma =G\delta_{t}/h$$

where τ is the shear stress and *G* is the shear modulus of the elastomer.

The portion of the total tangential force sustained by a single microcylinder can be expressed as:

(6) $$f_{t}=\tau A=G\frac{A}{h}\delta_{t}$$

(7) $$F_{t}=N\frac{GA}{h}\delta_{t}$$

where *N* represents the total number of microcylindrical elements.

Following tangential deformation, the inter-electrode spacing becomes $d=d_{0}-\delta_{t}$. Based on the capacitance formulation for parallel-plate structures, the functional relationship between the tangential force $F_{x}$ and the resulting capacitance change can be expressed as:

(8) $$F_{x}=\frac{NGA}{h}\left(d_{0}-\frac{\varepsilon_{0}\varepsilon_{r}S}{C_{F_{x}}}\right)$$

When the sensor is subjected to a bending moment $M_{x}$, the initial electrode spacing is denoted as $d_{0}$, and the electrode undergoes a rotation characterized by an angle $\theta_{x}$. Under this condition, the modified electrode spacing can be expressed as:

(9) $$d=d_{0}+y\cdot \tan\theta_{x}\approx d_{0}+y\cdot \theta_{x}$$

where *y* denotes the position coordinate along the electrode width.

Denoting the electrode width and length as W and L, respectively, the local capacitance contribution can be formulated in differential form. By integrating over the entire electrode surface, the total capacitance is obtained as:

(10) $$dC=\frac{\varepsilon_{0}\varepsilon_{r}L}{d_{0}+y\theta_{x}}dy$$

(11) $$C_{M_{x}}=\int_{-W/2}^{W/2}\frac{\varepsilon_{0}\varepsilon_{r}L}{d_{0}+y\theta_{x}}dy=\frac{\varepsilon_{0}\varepsilon_{r}L}{\theta_{x}}\ln\left(d_{0}+\frac{W}{2}\theta_{x}/d_{0}-\frac{W}{2}\theta_{x}\right)$$

where $\varepsilon_{0}$ is the vacuum permittivity, $\varepsilon_{r}$ is the relative permittivity of the dielectric layer.

On the basis of the established strain energy density formulation, the stress response and resulting compressive deformation of the microcolumn are derived as:

(12) $$\sigma_{z}=\frac{\partial W}{\partial \lambda }\cdot \lambda$$

The stretch ratio along the height direction is:

(13) λ(y)=h+Δh(y)/h=1−θy/h

Substituting $\lambda (y)=1-\theta_{y}/h$ into the Yeoh hyperelastic model leads to the position-dependent stress expression $\sigma_{z}(y)=\sigma_{\mathrm{Yeoh}}\lambda (y)$. Integrating this stress distribution along the microcolumn height results in:

(14) $$M_{x}=\int_{-b/2}^{b/2}\sigma_{z}(y)\cdot y\cdot ady=a\int_{-b/2}^{b/2}\sigma_{Yeoh}\left(1-\frac{\theta y}{h}\right)\cdot ydy$$

The same applies to My.

When the sensor is subjected to a torsional moment *M_z_*, a shear strain field is generated within the microcolumns, which can be expressed as:

(15) $$\gamma (r)=\frac{r\theta }{h_{0}}\left(0\leq r\leq R\right)$$

where *r* is the radial coordinate, *R* is the radius of the microcolumn, *h*_0_ is the initial height of the microcolumn, and *θ* is the torsional rotation angle.

Substituting this strain distribution into the Yeoh constitutive model yields the corresponding shear stress field τ(r).

(16) $$\tau (\gamma )=2C_{10}\gamma +4C_{20}\gamma^{3}+6C_{30}\gamma^{5}$$

In continuum mechanics, the torsional moment is defined as the surface integral of the shear stress weighted by its moment arm over the cross section, given by:

(17) $$M_{z}=\int_{0}^{R}2\pi r\cdot \tau (\gamma )\cdot rdr=\frac{\pi R^{4}\theta }{2h_{0}}\left[C_{10}+\frac{8C_{20}R^{2}\theta^{2}}{3h_{0}^{2}}+\frac{9C_{30}R^{4}\theta^{4}}{2h_{0}^{4}}\right]$$

The spacing between the lower parallel-plate sensing electrodes and the corresponding capacitance value can be expressed as:

(18) $$d(y,\theta )=d_{0}+y\tan\theta$$

(19) $$C_{M_{z}}=\varepsilon \varepsilon_{0}W\int_{0}^{H}\frac{dy}{d_{0}+y\tan\theta }=\frac{\varepsilon \varepsilon_{0}W}{\tan\theta }\ln\left(1+\frac{H\tan\theta }{d_{0}}\right)$$

Based on the established physical model, a Physics-Informed Neural Network (PINN) model has been developed in this study to decouple the capacitance signals into six-dimensional force/torque outputs. The input features consist of six capacitance values, while the hidden layer is composed of 128 neurons activated by the ReLU function. The output layer produces six values corresponding to three force components and three moment components.

In formulating the loss function, it is essential to compare the model’s predicted loading outputs with the actual loading outputs. This comparison assesses the model’s accuracy in predicting forces and moments, leading to a quantifiable measure of data loss:

(20) $$L_{data}=\sum_{i=1}^{6}{\left\|F_{pred}^{i}-F_{true}^{i}\right\|}^{2}$$

*F_pred_* denotes the load predicted by the model, while *F_true_* represents the actual applied load.

Unlike purely data-driven approaches, Physics-Informed Neural Networks (PINNs) incorporate governing physical laws directly into the loss function through a physics-based residual term. This integration is crucial for enhancing generalization and ensuring physical consistency, particularly for problems with limited or noisy data, where physics serves as the most reliable source of constraints. The physical loss is calculated based on the Mean Squared Error between *F_phy_* and *F_pred_*, we obtain:

(21) $$L_{phys}=\sum_{i=1}^{6}{\left\|F_{pred}^{i}-F_{phy}^{i}\right\|}^{2}$$

Here, *F*_phy_ denotes the theoretical load derived from the physical model, which is employed to formulate the physics-informed residual.

Limitations associated with boundary conditions are analyzed by considering shape variables, specifically displacement d and rotation angle *θ*, as the primary predictors. When a known force *F_i_* or moment *M_i_* is applied at the structure’s boundary, subject to specified constraints on electrode displacement and angular rotation, a corresponding global response-either a translational displacement *d_i_* or rotational angle *θ_i_*-can be observed at the apex of the structure. Examples of such boundary conditions include:

(22) $$L_{F_{BC}}=\sum_{i\in \left\{x,y,z\right\}}{\left(f_{d}\left({\hat{F}}_{i}\right)-d_{i}\right)}^{2}$$

(23) $$L_{M_{BC}}=\sum_{i\in \left\{x,y,z\right\}}{\left(f_{\theta }\left({\hat{M}}_{i}\right)-\theta_{i}\right)}^{2}$$

The associated loss function is.

(24) $$L_{BC}=L_{F_{BC}}+L_{M_{BC}}$$

Combining the above loss functions, the total loss function is:

(25) $$L_{Loss}=\lambda_{data}L_{data}+\lambda_{phy}L_{phy}+\lambda_{BC}L_{BC}$$

**Figure 5:**
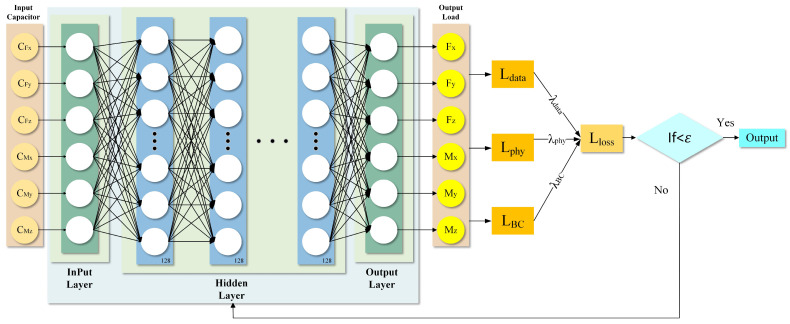
PINN training model.

The equilibrium between data fitting and adherence to physical constraints is regulated by adjusting the weights of λ_data_, λ_phy_, and λ_BC_. TensorFlow autonomously computes the gradient and modifies the model’s weights through the optimizer and loss function. Practically, increasing λ_data_ enhances the network’s fidelity to measured data, increasing λ_phy_ enforces stronger physical consistency, while increasing λ_BC_ improves boundary conformity. The architecture of the neural network is illustrated in Figure 5.

## 4. Assessment and Evaluation of Sensors

### 4.1. Evaluation of Sensor Performance

To investigate the sensing performance, decoupling accuracy, and practical application capability of the proposed sensor, three experimental setups were designed.

**Figure 6:**
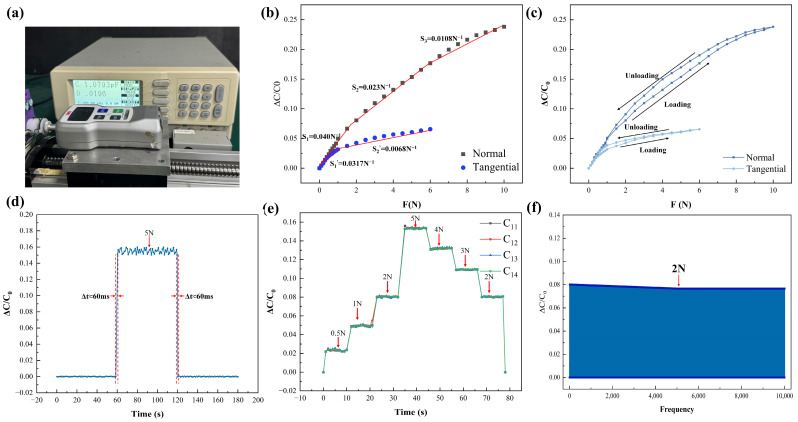
Evaluation of sensor performance: (**a**) data acquisition system; (**b**) diagram illustrating the sensor’s sensitivity at each loading period; (**c**) sensor hysteresis graph; (**d**) sensor reaction time; (**e**) dynamic loading efficacy of the sensor; (**f**) capacitance retention.

As is shown in Figure 6a, Sensor performance was evaluated on a servo motor–driven ball screw test platform. Loads were applied using a push–pull force gauge (FGJ-5, SHIMPO, Kyoto, Japan) with a custom fixture. Capacitance measurements were conducted with a precision LCR meter (LCR−821, GWINSTEK, New Taipei City, Taiwan, China) at 100 kHz and 1V excitation.

The second experiment was designed for the decoupling analysis under multi-dimensional loading conditions. The decoupling experiments were conducted using a six-axis loading platform. The experimental platform is presented in Figure S2a, while its operating principle and the designed loading mold are shown in Figure S2b and Figure S2c, respectively.

Figure S2b illustrates that the experimental platform simulates six-dimensional force loading by applying weights to a mold through a fixed pulley system. Pulley 1 applies a calibration force in the x-direction, pulley 2 in the *y*-direction, and pulley set 3 produces a calibration moment about the Mx-axis. Other directional forces and moments are applied in a similar manner. The calibration setup allows simultaneous application of forces and moments in multiple directions, facilitating the testing of combined loading conditions.

**Figure 7:**
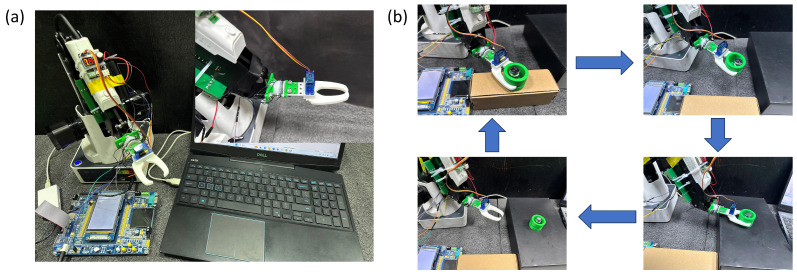
Experimental performance evaluation of sensor grasping; (**a**) hardware and software of the sensor and its grasping system; (**b**) operation modes and workflow of the robotic arm.

The third experiment was conducted to verify the sensor’s performance in a practical working environment. As shown in Figure 7, the experimental setup consists of a multifunctional robotic arm, a mechanical gripper, a control unit, and a signal acquisition circuit. The acquisition circuit integrates a microcontroller with an AD7147-1 (AD7147-1, Analog Devices, Wilmington, MA, USA) capacitive-to-digital converter, which features on-chip automatic calibration to compensate for environmental variations such as temperature drift and parasitic capacitance fluctuations. This design ensures stable and reliable measurement performance of the six-axis force sensor under dynamic operating conditions.

### 4.2. Experimental Evaluation of Sensor Performance

The mechanical testing setup is shown in Figure 6a. Under axial loading (Figure 6b), the sensor exhibited sensitivities of 0.0403 N^−1^ in the 0–2 N range, 0.023 N^−1^ in the 2–8 N range, and 0.0108 N^−1^ in the 8–10 N range. For tangential loading, sensitivities were 0.0317 N^−1^ within 0–1 N and 0.0068 N^−1^ within 1–6 N. Hysteresis performance was characterized through loading–unloading cycles (Figure 6c), yielding hysteresis rates of 4.7% for normal force and 1.26% for tangential force. Dynamic response characteristics are presented in Figure 6d. Under a 5 N axial load, the relative capacitance change increased to approximately 0.16 within 60 ms and subsequently reached a stable state. A durability test involving 10,000 loading cycles at 2 N (Figure 6f) showed approximately 98% capacitance retention, indicating stable long-term performance. Figure S1b–d show that, under both normal and tangential loading conditions, all sensing units exhibit consistent response characteristics, with closely aligned variation trends. This behavior reflects the structural symmetry of the sensor and the coordinated deformation among sensing elements. Figure S1e further indicates that, during repeated loading cycles, the output signals of all sensing units remain stable, with no noticeable attenuation or drift. These results demonstrate good repeatability and operational stability of the sensor.

Overall, the results demonstrate stable sensitivity, moderate hysteresis, fast response, and reliable cyclic durability under both normal and tangential loading conditions.

### 4.3. Comparison and Analysis of Errors Based on the PINN Decoupling Algorithm

Based on the experimental loading system shown in Figure S2a, different combinations of six-dimensional loads were applied, and a total of 300 sets of corresponding capacitance data were collected. During the data acquisition process, combined loading conditions were applied using the six-axis loading platform, where different combinations of forces and moments were introduced simultaneously. The loading configurations were designed to span the operational ranges of $F_{x}$, $F_{y}$, $F_{z}$, $M_{x}$, $M_{y}$, and $M_{z}$, enabling the dataset to adequately cover the six-degree-of-freedom load space. As a result, the trained model primarily performs interpolation within the sampled load domain, thereby reducing the risk of extrapolation during testing.

The collected 300 calibration samples were used for model training. In addition, 16 sets of capacitance data that were not involved in the training process were reserved as an independent test set to evaluate the prediction performance of the decoupling model. Since the dataset was generated through controlled loading experiments using the six-axis calibration platform and the samples cover the operational load ranges of the sensor, the independent test set provides a reliable evaluation of the model performance under unseen loading conditions.

To enable a clear calibration and analysis of the errors associated with the proposed PINN decoupling algorithm, several representative decoupling algorithms were selected for comparison, including polynomial regression, backpropagation (BP) neural networks, feedforward networks (FFN), and Residual Networks (ResNet).

All baseline models (polynomial regression, BP, FFN, and ResNet) were trained using the same input features and the same dataset as the proposed method to ensure a fair comparison. The measured capacitance signals were used as inputs and the corresponding six-axis force/torque components as outputs. All models adopted the same training–validation split and comparable training strategies.

**Figure 8:**
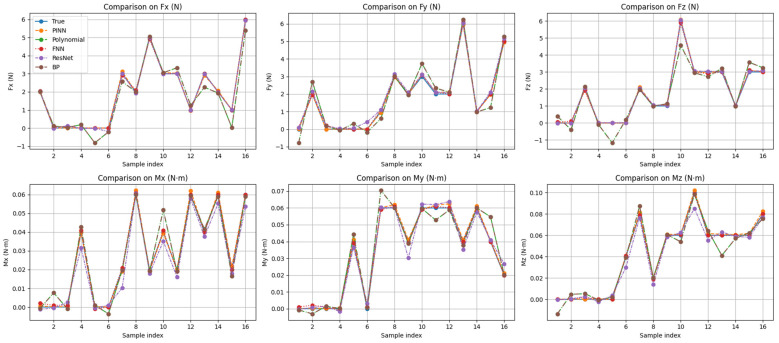
Error analysis graph of neural network methods exhibiting various architectures.

As is shown in Figure 8, Compared with conventional decoupling algorithms, the proposed PINN-based decoupling method demonstrates superior accuracy in six-dimensional force measurements. The average relative errors are 1.75%, 1.20%, and 1.31% for the force components ($F_{x}$, $F_{y}$, and $F_{z}$), and 0.95%, 0.93%, and 0.97% for the moment components ($M_{x}$, $M_{y}$, and $M_{z}$), respectively.

An ablation study was performed to evaluate the effectiveness of the proposed PINN. As shown in Table 2, both the linear mapping method and the purely data-driven neural network exhibit larger prediction errors, indicating their limitations in capturing the nonlinear coupling characteristics of the sensor. By incorporating physical constraints into the learning process, the proposed PINN achieves significantly improved accuracy and robustness. This result confirms the effectiveness of integrating physical constraints into the neural network for enhancing the decoupling accuracy.

The ablation results indicate that the linear mapping model exhibits relatively large errors due to its limited ability to capture the nonlinear coupling among different force components. Although the purely data-driven neural network improves the prediction in some channels, it still shows significant instability in others. In contrast, the proposed PINN achieves consistently lower errors across all channels, demonstrating that the incorporation of physics-based constraints plays the dominant role in improving the decoupling accuracy.

To further evaluate the robustness of the Physics-Informed Neural Network (PINN) algorithm, small-sample training experiments were designed to test the model’s stability under conditions of limited data. As shown in Table 3, when the training dataset is small, the PINN-based method maintains a relatively high level of decoupling accuracy, thereby demonstrating its robustness.

In conclusion, the proposed PINN method effectively integrates physical priors into the learning process. This integration ensures mechanical consistency and enhances the model’s robustness, accuracy, and interpretability, even when operating under small-sample conditions.

It should be noted that the use of flexible polymer materials may introduce hysteresis during the loading and unloading processes, which can lead to slightly different sensor outputs under the same external load and increase the difficulty of learning the input–output relationship. In this study, the calibration and data acquisition procedures were mainly conducted during the loading stage, which represents the primary operating condition of the sensor in practical measurements. Under this experimental strategy, the influence of hysteresis on the collected dataset is relatively limited. In addition, the proposed PINN incorporates physical constraints, which improves the stability and generalization capability of the model and further reduces the impact of hysteresis on the regression results.

### 4.4. Evaluation and Testing of Sensor Decoupling Applications

After evaluating the sensing performance on the loading platform, the sensor was further tested in a practical working environment to demonstrate its application capability.

Figure 7 illustrates the experimental setup used for the practical application test along with its working process. The experimental platform comprises a multifunctional robotic arm, a mechanical gripper, a control unit, and a signal acquisition circuit. This circuit integrates a microcontroller with an AD7147-1 capacitive-to-digital converter. Featuring on-chip automatic calibration, the AD7147-1 continuously compensates for subtle environmental variations, including temperature drift and parasitic capacitance fluctuations. As a result, the six-axis force sensor delivers consistent and dependable performance under dynamic operating conditions.

**Figure 9:**
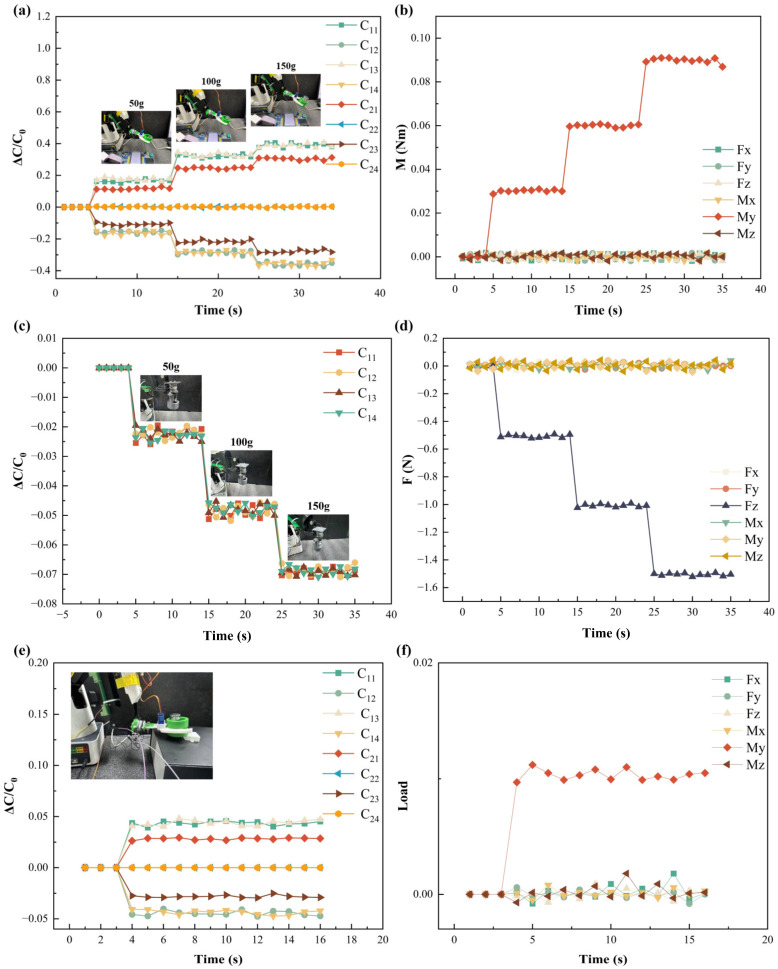
Experimental results of sensor load testing: (**a**) experimental measurements obtained from the hierarchical grasping sensor; (**b**) torque estimation results based on the proposed decoupling model; (**c**) sensor responses under vertical mounting configuration; (**d**) decoupled normal force estimation; (**e**) sensor outputs under abnormal grasping conditions; (**f**) identification of interference torques through the decoupling model.

To evaluate the sensor’s ability to distinguish objects with different masses, samples of 50 g, 100 g, and 200 g were sequentially placed on the gripper. The sensor was tested under different mounting orientations. As shown in Figure 9a, when installed parallel to the ground, the sensor clearly differentiated the three loading conditions. The measured signals were input into the decoupling model, yielding the estimated torques shown in Figure 9b, with average values of 0.0354 N·m, 0.065 N·m, and 0.12 N·m, respectively. These results agree well with the theoretical values, indicating accurate torque estimation.

When mounted vertically on the robotic arm, the sensor primarily responded to normal forces. The response curves under different weights are presented in Figure 9c. After decoupling, the predicted forces were approximately 0.6 N, 1 N, and 2 N as is shown in Figure 9d, consistent with the applied loads, thereby validating the effectiveness of the proposed decoupling method.

As shown in Figure 9e, when the object deviates from the designated horizontal plane, the lower sensing units (C_21_–C_24_) generate torque-related signals. The corresponding decoupled outputs are presented in Figure 9f, demonstrating the detection of interference torques. By comparing real-time outputs with calibrated reference values, placement deviations can be effectively identified, enabling stable operational monitoring.

## 5. Conclusions

In summary, this paper presents a rigid–flexible coupled capacitive six-dimensional force sensor. Through the synergistic design of a flexible sensing unit array and a rigid support structure, the proposed sensor enables directional load perception and achieves preliminary decoupling at the structural level. This structural pre-decoupling establishes a clear mathematical relationship between external loads and the outputs of individual sensing units, providing a solid foundation for subsequent model-based decoupling. However, due to the inherent coupling characteristics of the sensor structure, complete decoupling cannot be achieved solely through mechanical design. To address this limitation, a Physically Informed Neural Network (PINN) is introduced to further optimize decoupling at the algorithmic level. By embedding a silicone deformation model as a physical constraint into the training process, the proposed method achieves high-precision decoupling even with a limited training dataset. Experimental results demonstrate that the sensor attains full-scale sensing accuracies of 99.3%, 98.8%, and 98.6% for force measurements, with corresponding decoupling errors of 1.75%, 1.20%, and 1.31%. For torque measurements, full-scale sensing accuracies of 98.7%, 98.5%, and 98.0% are achieved, with decoupling errors of 0.95%, 0.93%, and 0.97%. These results validate the effectiveness of the proposed integrated “structural design–neural network” decoupling strategy. Furthermore, when deployed on a multifunctional robotic arm, the proposed sensor accurately identifies external loads, detects abnormal grasping postures of the end-effector, and enables preliminary assessment of the robotic arms operational state based on multi-dimensional sensing outputs. This work provides a solid foundation for the development of intelligent grasping systems and integrated sensor–robot motion control frameworks.

## Figures and Tables

**Table 1:** Decoupling principles of sensors.

Force	C_11_	C_12_	C_13_	C_14_	C_21_	C_22_	C_23_	C_24_
F_x_	-	-	-	-	↑	-	↓	-
F_y_	-	-	-	-	-	↑	-	↓
F_z_	↑	↑	↑	↑	-	-	-	-
M_x_	↓	↓	↑	↑	-	↑↑	-	↓↓
M_y_	↑	↓	↑	↓	↑↑	-	↓↓	-
M_z_	-	-	-	-	↑	↓	↑	↓

In this table, “↑” denotes an increase in the capacitance value of a sensing unit, while “↓” denotes a decrease. “↑↑” indicates an increase in the capacitance value caused by structural coupling, whereas “↓↓” indicates a decrease caused by structural coupling.

**Table 2:** Ablation study results of different decoupling methods.

	*F*_x_	*F*_y_	*F*_z_	*M*_x_	*M*_y_	*M*_z_
PINN	1.75%	1.2%	1.31%	0.95%	0.93%	0.97%
Linear mapping	14.2%	15.89%	8.69%	6.49%	8.03%	7.01%
PINN without BC	3.7%	2.91%	2.87%	2.13%	3.0%	2.45%
NN	10.2%	49.7%	11.3%	23.9%	21.7%	5.9%

**Table 3:** Verification of decoupling algorithm robustness testing.

	*F*_x_	*F*_y_	*F*_z_	*M*_x_	*M*_y_	*M*_z_
10% PINN	66.2%	46.1%	9.8%	22.3%	19.5%	14.2%
10% BP	72.7%	53.8%	12.5%	27.1%	24.4%	21.0%
10% RestNet	715%	49.7%	11.3%	23.9%	21.7%	17.8%
30% PINN	16.4%	12.7%	3.1%	11.25%	7.62%	10.32%
30% BP	32.1%	21.5%	5.5%	18.12%	9.35%	8.89%
30% RestNet	27.9%	18.3%	4.8%	21.5%	8.26%	7.06%
50% PINN	2.04%	7.05%	2.11%	5.5%	4.9%	3.8%
50% BP	5.05%	8.30%	3.15%	10.2%	6.8%	6.6%
50% RestNet	4.25%	5.10%	4.15%	8.3%	6.7%	5.9%

## Data Availability

The data presented in this study are available on request from the corresponding author.

## Supplementary Materials

The following supporting information can be downloaded at: https://www.mdpi.com/article/10.3390/s26072038/s1, Figure S1: Diagram of the sensor’s general construction and loading characteristics. (a) Experimental loading slide (b) Sensor normal loading characteristics (c) Sensor loading characteristics in the x-direction (d) Sensor loading characteristics in the y-direction (e) Sensor characteristics under recurrent loading. Figure S2: Sensor loading platform (a) Structural diagram of the loading platform. (b) Diagram illustrating the operational principle of the loading platform (c) Schematic representation of the integrated functionality of the mold sensors.
