# Supplementary material for: A Rigid–Flexible Coupled Six-Dimensional Force Sensor and Its PINN-Based Decoupling Algorithm
Source: Sensors (Basel). 2026 Mar 25;26(7):2038. doi: 10.3390/s26072038 (PMC13074577; doi:10.3390/s26072038)
Supplement: Supplementary file 1 [file sensors-26-02038-s001.zip › sensors-4182779-supplementary.pdf]

Supplementary material

.

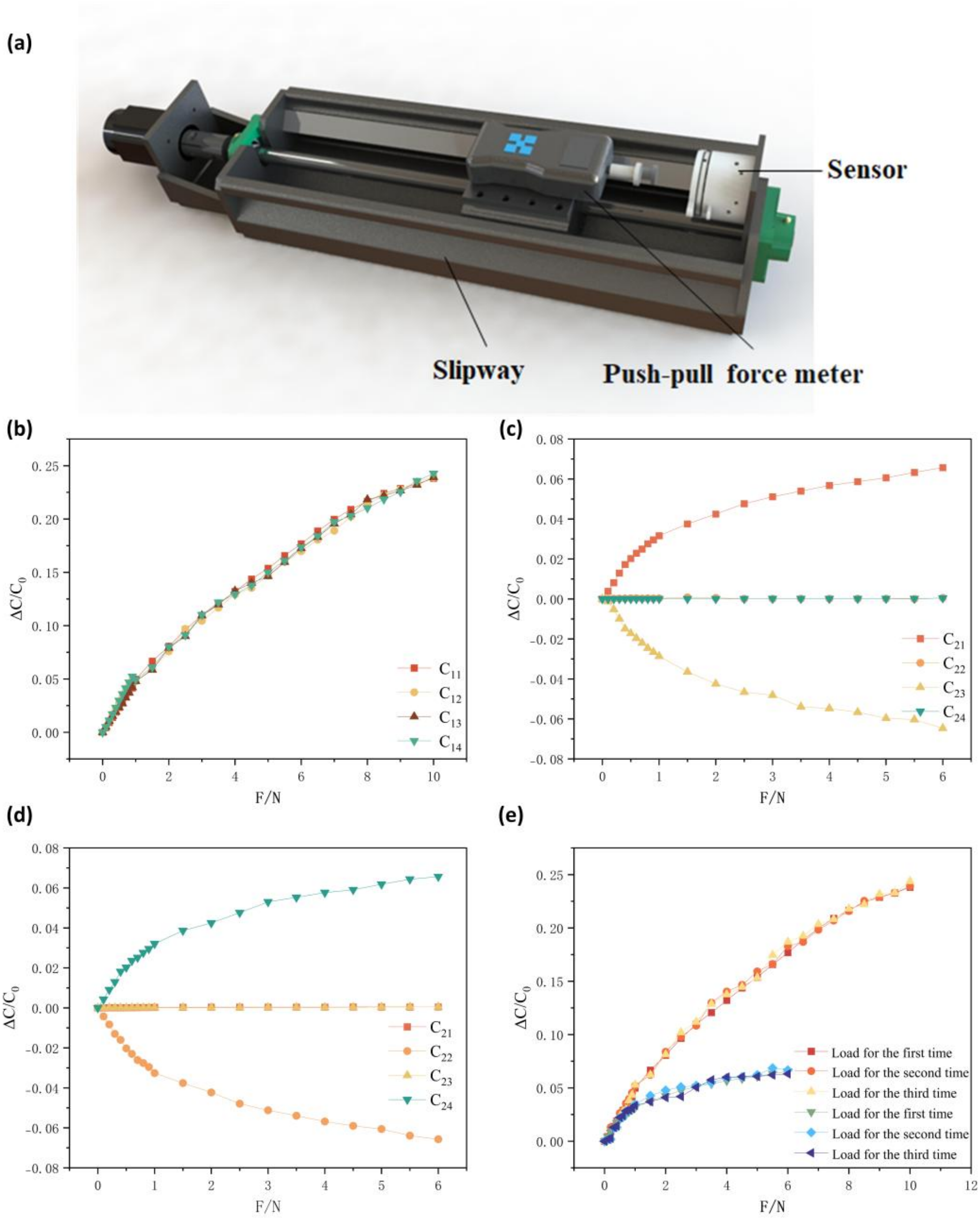

Figure S1. Diagram of the sensor's general construction and loading characteristics. (a) Experimental loading slide (b) Sensor normal loading characteristics (c) Sensor loading characteristics in the x-direction (d) Sensor loading characteristics in the y-direction (e) Sensor characteristics under recurrent loading

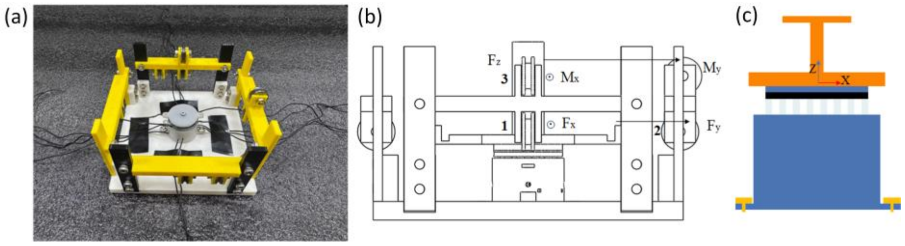

Figure S2. Sensor loading platform (a) Structural diagram of the loading platform. (b) Diagram illustrating the operational principle of the loading platform (c) Schematic representation of the integrated functionality of the mold sensors
